# Enhancing immunotherapy efficacy with synergistic low-dose radiation in metastatic melanoma: current insights and prospects

**DOI:** 10.1186/s13046-025-03281-2

**Published:** 2025-01-30

**Authors:** Zahid Rafiq, Mingyo Kang, Hampartsoum B. Barsoumian, Gohar S. Manzar, Yun Hu, Carola Leuschner, Ailing Huang, Fatemeh Masrorpour, Weiqin Lu, Nahum Puebla-Osorio, James W. Welsh

**Affiliations:** 1 Department of Radiation Oncology, The University of Texas MD Anderson Cancer Center, Houston, TX 77030 USA; 2 Department of Pharmaceutical Sciences, School of Pharmacy, University of Texas at El Paso, 500 W. University Ave, El Paso, TX 79968 USA

## Abstract

Recent advances in oncology research have highlighted the promising synergy between low-dose radiation therapy (LDRT) and immunotherapies, with growing evidence highlighting the unique benefits of the combination. LDRT has emerged as a potent tool for stimulating the immune system, triggering systemic antitumor effects by remodeling the tumor microenvironment. Notably, LDRT demonstrates remarkable efficacy even in challenging metastatic sites such as the liver (*uveal*) and brain (*cutaneous*), particularly in advanced melanoma stages. The increasing interest in utilizing LDRT for secondary metastatic sites of uveal, mucosal, or cutaneous melanomas underscores its potential efficacy in combination with various immunotherapies. This comprehensive review traverses the journey from laboratory research to clinical applications, elucidating LDRT’s immunomodulatory role on the tumor immune microenvironment (TIME) and systemic immune responses. We meticulously examine the preclinical evidence and ongoing clinical trials, throwing light on the promising prospects of LDRT as a complementary therapy in melanoma treatment. Furthermore, we explore the challenges associated with LDRT’s integration into combination therapies, addressing crucial factors such as optimal dosage, fractionation, treatment frequency, and synergy with other pharmacological agents. Considering its low toxicity profile, LDRT presents a compelling case for application across multiple lesions, augmenting the antitumor immune response in poly-metastatic disease scenarios. The convergence of LDRT with other disciplines holds immense potential for developing novel radiotherapy-combined modalities, paving the way for more effective and personalized treatment strategies in melanoma and beyond. Moreover, the dose-related toxicities of immunotherapies may be reduced by synergistic amplification of antitumor efficacy with LDRT.

## Introduction

In the landscape of advanced melanoma research, both radiation therapy and immunotherapy have ushered in a transformative era in treatment strategies. Immunotherapy has revolutionized the approach to melanoma treatment by using the body’s immune system to target and eradicate metastatic lesions, offering newfound optimism to both patients and healthcare providers. Melanoma, originating from pigment-producing cells known as melanocytes, manifests in various forms, including cutaneous, mucosal, and ocular melanomas. In its advanced stages (stages 3 and 4), the disease disseminates to distant sites such as lymph nodes, lungs, liver, bones, and brain, culminating in metastatic melanoma [1]. Approximately 90% of uveal melanoma metastasizes to the liver, while cutaneous melanoma has the potential to disseminate to various locations, including lymph nodes, lungs, brain, and soft tissue. According to the American Cancer Society (ACS), an estimated 100,640 adults in the United States will receive a melanoma diagnosis in 2024. The American Society of Clinical Oncology (ASCO) reports that around 4% of individuals are diagnosed with melanomas that have progressed to distant sites, representing the most advanced stage of metastatic melanoma. Data from the National Cancer Institute [2] further corroborates these findings. Approximately 8.5% of individuals diagnosed with melanoma experience spread to nearby lymph nodes, and these cases typically carry a somewhat improved prognosis. Uveal melanoma, characterized by its rarity and insidious nature, presents an incidence rate of 4.6 per million. Unfortunately, metastatic uveal melanoma (mUM) is associated with a bleak prognosis, with a 2-year overall survival rate of only 8% [3–7].

**Fig. 1:**
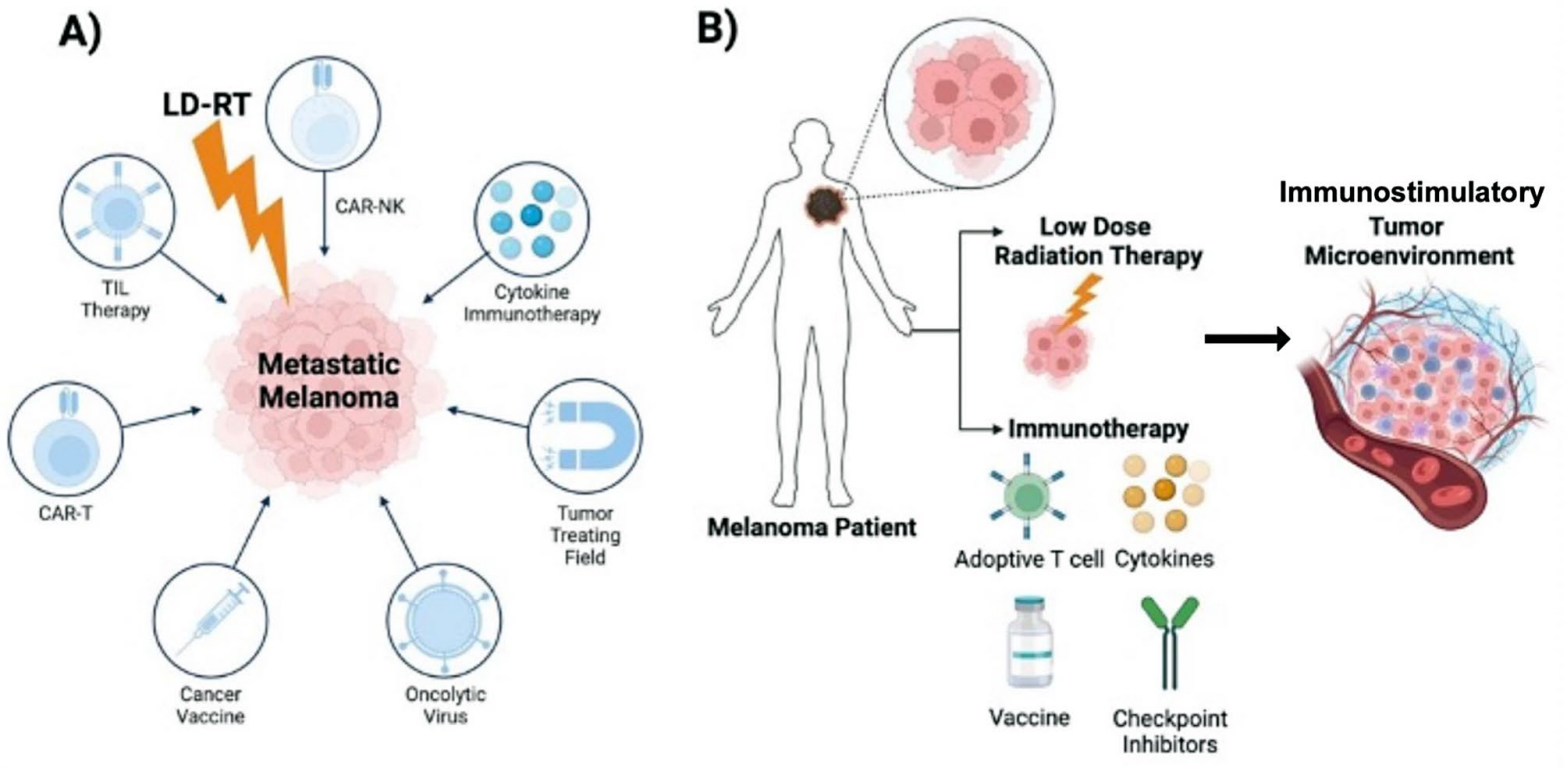
Low-dose radiation therapy (LDRT) combinations with immunotherapies in metastatic melanomas. **A** Overview of various immunotherapies, including the most recent and clinically relevant strategies, combined with LDRT for treating metastatic melanoma. These combinations explore synergies between LDRT and immunotherapeutic approaches to enhance therapeutic efficacy. **B** The combination of LDRT with immunotherapies amplifies the antitumor immune response by reprogramming the tumor immune microenvironment (TIME), shifting it from immunosuppressive to immunostimulatory, thereby targeting metastatic lesions effectively

Most patients with metastatic uveal melanoma do not achieve favorable outcomes with immunotherapy, unlike those with cutaneous melanoma, which has a five-year overall survival rate of 50%. This disparity is primarily due to several factors, including poor antigen presentation, a low mutational burden, limited neo-antigenicity, deficient T-cell priming and infiltration, T-cell exhaustion, and the suppressive nature of the tumor stroma [4, 8–10]. These factors collectively impede the function and entry of immune effector cells, contributing to the challenges encountered in achieving successful treatment responses [4, 11–13]. Hence, it is imperative to devise strategies aimed at sensitizing tumors, effectively transforming “cold” or inert tumors into “hot” tumors, to bolster responses to immunotherapy, particularly in cases that have shown non-responsiveness or recurrence. Low-dose radiation therapy (LDRT) has demonstrated the capacity to transiently induce inflammation within tumors, rendering them more conducive and receptive to immunotherapy across a spectrum of malignancies [3, 14–16]. Several types of immunotherapies, including novel approaches like tumor-treating fields and oncolytic viruses, have been tested with LDRT (Fig. 1A).

LDRT, typically administered at doses between 0.5 Gy and 2 Gy, enhances the efficacy of immunotherapies, such as immune checkpoint inhibitors (ICI) or cell therapy, by altering immunosuppressive factors. Recent compelling studies have highlighted LDRT’s significant role in reshaping and modulating the tumor immune microenvironment, as illustrated in Fig. 1B. This modification empowers immune effector cells to launch robust responses against cancer cells [3, 14, 17, 18].

High-dose radiation therapy (HDRT) initiates an in-situ tumor vaccination effect (ISV), prompting systemic antitumor immunity and the noteworthy abscopal effect, wherein distant non-irradiated organs experience immune-mediated destruction of metastatic lesions. However, using HDRT poses risks to both normal surrounding tissues and the viability of tumor-infiltrating lymphocytes (TILs). While radiation therapy can modulate the antitumor immune response, the effective radiation dose to immune cells (EDIC) in circulating blood is pivotal for tumor control. Reducing radiation exposure to the immune system holds promise for enhancing survival outcomes. Thus, there is a growing interest in using LDRT owing to its ability to strengthen anticancer immune responses when combined with immunotherapies [19, 20]. Several prospective studies underscore the increasing significance of LDRT in the context of metastatic melanoma. Notably, LDRT applied to liver metastases has demonstrated a significant enhancement in the immunotherapeutic effectiveness of a dual regimen comprising PD-L1 (atezolizumab) and VEGFA blockade (bevacizumab). This augmentation is attributed to the mobilization of CD8+ T cells into the tumor, facilitated by the CXCL10/CXCR3 axis, mainly observed in hepatocellular carcinoma (HCC) [14]. In advanced uveal melanoma, liver metastases predominate and often exhibit resistance to immunotherapy. However, an intriguing clinical observation arose from a stage IV melanoma patient who achieved a complete response following low-dose radiation therapy (LDRT) targeting liver metastases. This patient received a regimen of 5.6 Gy administered over four fractions (1.4 Gy per fraction), encompassing nearly the entire liver [21]. This case highlights that hepatic metastases, typically resistant due to their immunologically cold nature, can be rendered susceptible to treatment through LDRT.

In preclinical models of immunologically cold melanoma, low-dose whole-brain radiation therapy (LD-WBRT) at 4 Gy in a single fraction significantly reduced intracranial tumors and improved survival rates. Conversely, a high-dose radiation-based in-situ vaccination (ISV) regimen, incorporating 12 Gy in a single fraction, immune-cytokines, and anti-CTLA-4, effectively eradicated primary flank tumors but demonstrated limited efficacy against intracranial tumors [22]. ISV strategies harness tumor tissue as a source of antigens to modulate immune responses and induce systemic antitumor immunity, making them particularly promising in overcoming the immunosuppressive microenvironments characteristic of uveal melanoma. In these settings, tumor cells evade antigen presentation and suppress T-cell activation; however, ISV reprograms the tumor microenvironment (TME) into an immunostimulatory state enriched with activated cytotoxic T-cells capable of attacking tumors.

These findings suggest that even challenging metastatic sites, such as brain metastases from advanced cutaneous melanoma, can be made vulnerable to treatment through LDRT. By reshaping the TME and augmenting immunotherapy responsiveness, LDRT holds potential across diverse forms of metastatic melanoma. This review synthesizes evidence supporting LDRT’s capacity to transform resistant tumor sites and advocates for its future integration with immunotherapeutic strategies in cancer treatment.

## Mechanisms induced by low-dose radiation therapy (LDRT) to foster an immune-inflamed tumor microenvironment augmenting the effectiveness of antitumoral immunotherapy

**Fig. 2:**
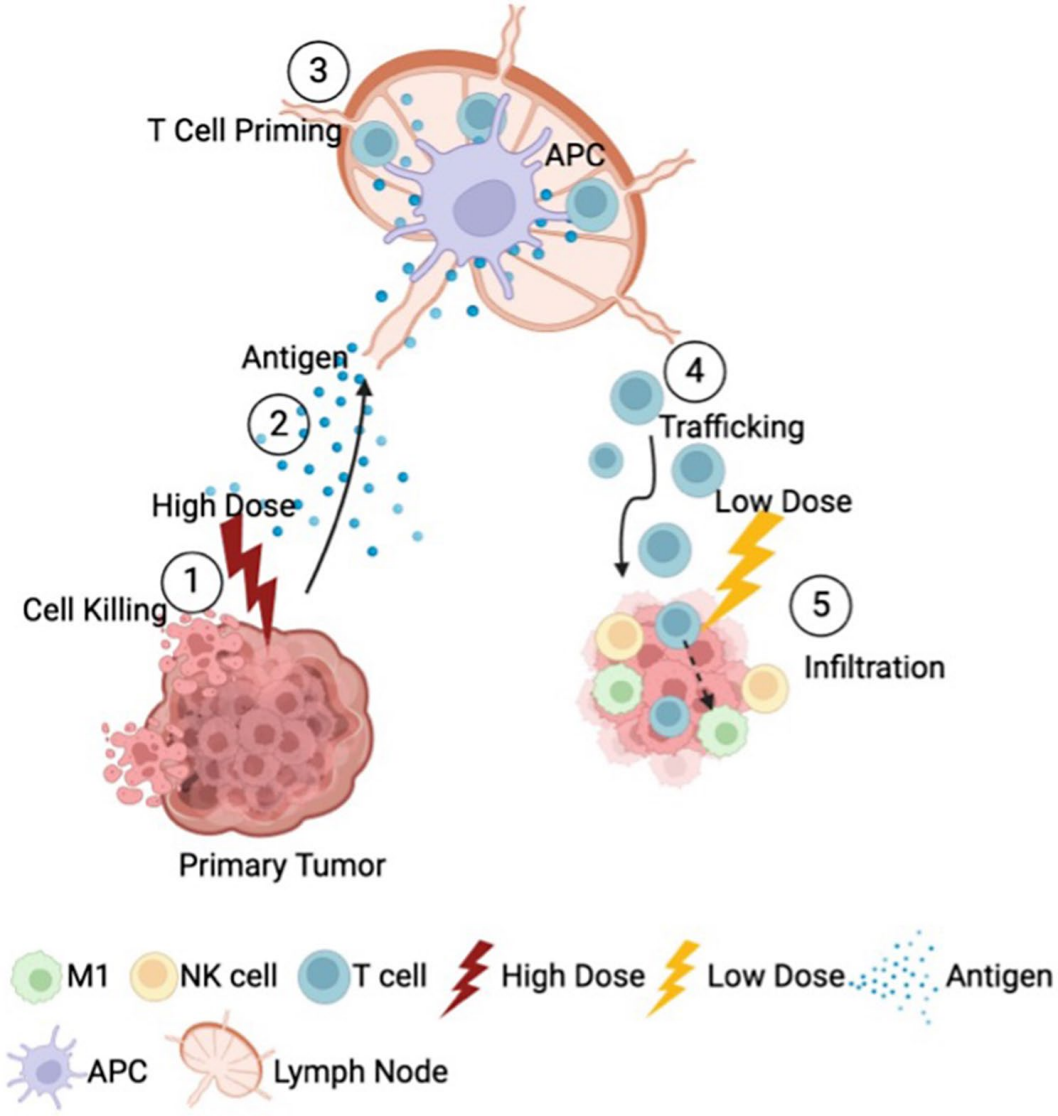
A visual representation of the immune cell cycle illustrating the effects of high-dose and low-dose radiation. (1) High-dose radiation effectively destroys primary tumor cells, (2) facilitating the release of antigens, and (3) initiating T-cell priming. (4) Immunotherapy reduces T-cell exhaustion and promotes lymphocyte movement to secondary tumor sites. (5) Low-dose radiation, on the other hand, affects the tumor stroma, enhancing the infiltration of natural killer (NK) cells and T cells into secondary tumor sites. This leads to better immune recognition of tumor cells and continuous tumor cell destruction with ongoing antigen release

The immunosuppressive landscape in solid tumors is characterized by a combination of physical and cellular barriers that inhibit effective anti-tumor immune responses. These include the presence of an inhibitory stroma, immunosuppressive cell populations such as regulatory T cells (T_regs_), myeloid-derived suppressor cells (MDSCs), and tumor-associated macrophages (M2 phenotype). Additionally, the tumor microenvironment (TME) is enriched with inhibitory cytokines like TGF-β, which collectively create a hostile environment that dampens cytotoxic T-cell activity and impairs the overall immune response against the tumor [3, 23, 24]. Like immuno-oncology agents, LDRT can profoundly remodel the TME, fostering conditions that enhance immune effector cell infiltration and promote tumor eradication by modulating the stromal components. LDRT induces systemic antitumor effects through a range of mechanisms, including the activation and recruitment of immune cells, increased cytokine production, redirection of immune responses toward an antitumor phenotype, modulation of gene expression profiles, and disruption of key immunosuppressive pathways within the TME. These multifaceted effects underscore LDRT’s potential to synergize with immunotherapies for improved cancer treatment outcomes [17, 25]. Due to its ability to enhance systemic antitumor effects, there has been increasing interest in using LDRT as a complementary approach to high-dose radiation therapy (HDRT), chemotherapy, immunotherapy, or cell therapy [26]. HDRT and chemotherapy primarily work by directly killing tumor cells, which stimulate the expression of MHC class I molecules within the tumor microenvironment (TME), a critical factor for immune recognition. This is particularly relevant in cases resistant to anti-PD1 therapy, which often exhibit reduced MHC class I expression. Additionally, HDRT facilitates the release of tumor neoantigens, essential for T-cell priming and diversification of the T-cell receptor (TCR) repertoire. When combined with LDRT, these effects can be further amplified, fostering a more robust and targeted immune response against tumors [27]. The immune response generated at oligometastatic sites far from the localized application of HDRT is termed the abscopal effect (Fig. 2). This phenomenon results in the regression of metastatic lesions distant from the irradiated site, showcasing the systemic potential of radiation therapy. However, HDRT can also induce adverse effects, such as T-cell exhaustion and the upregulation of immunosuppressive factors like TGF-β and regulatory T cells (T_regs_) [28].

**Fig. 3:**
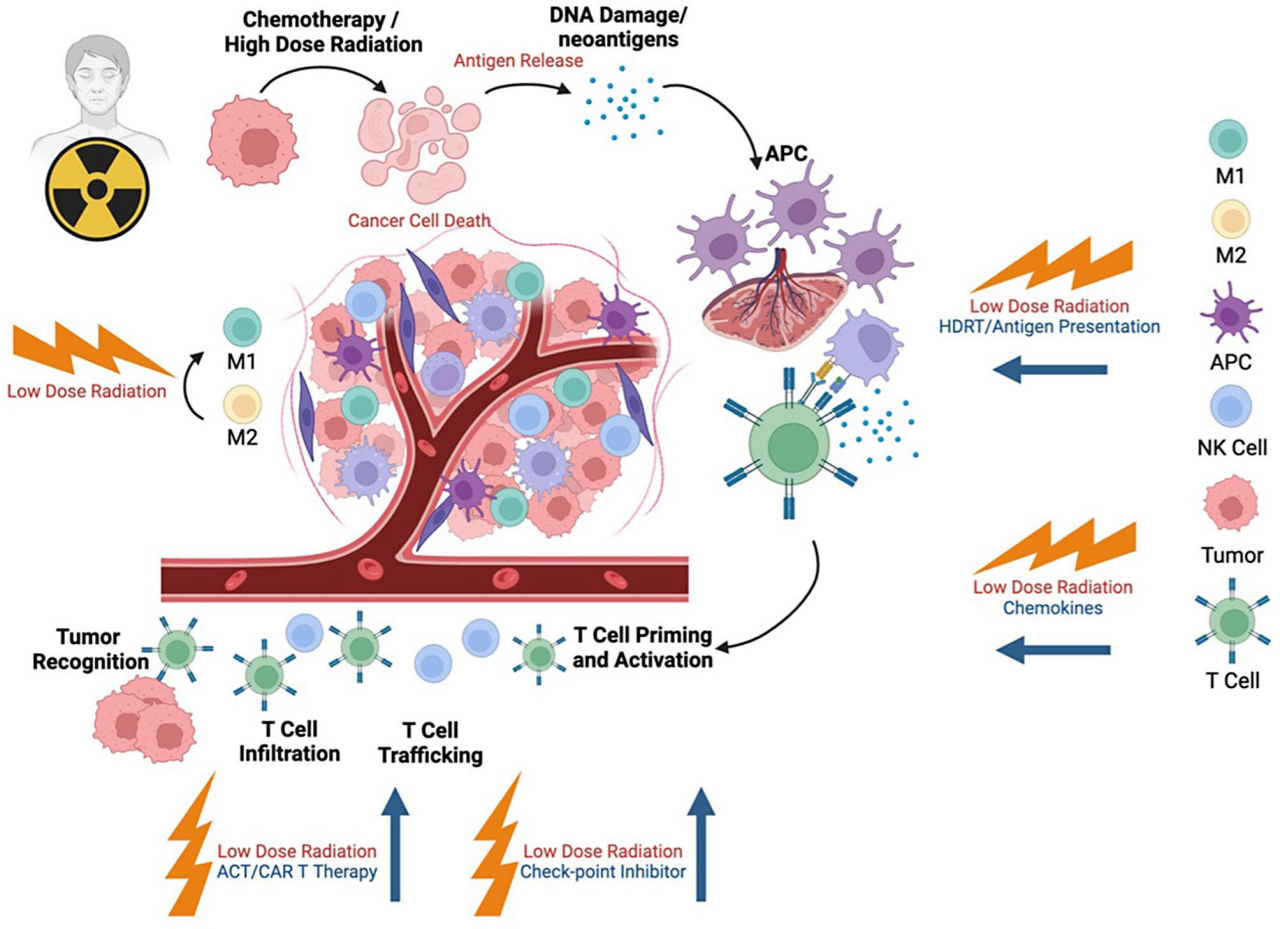
Illustration of the mechanism-based application of immunotherapies with LDRT. The release of tumor-associated antigens by high-dose radiation therapy (HDRT) or chemotherapy initiates T-cell priming, further amplified by enhanced T-cell infiltration to metastatic sites using low-dose radiation therapy (LDRT). At this stage, combining immunotherapies with LDRT can be particularly beneficial. The release of chemokines, stromal modulation, and further boosting of T cell priming enhance the effects of ICI (anti-PD1 plus anti-CTLA-4). Additionally, the increased infiltration of T and NK cells helps harness the synergistic effects of adoptive cell therapy (ACT) when combined with LDRT. Moreover, LDRT can convert M2 tumor-associated macrophages (TAMs) to the antitumor M1 type. This also explains the positive abscopal antitumor immune responses observed at metastatic sites when using LDRT combined with immunotherapies

A novel approach called the Radscopal Technique has been proposed to mitigate these drawbacks. This strategy combines HDRT at the primary tumor site with LDRT targeted at secondary metastatic sites. LDRT enhances the immune-stimulatory effects of HDRT by modulating the TME [24]. Specifically, it promotes the conversion of tumor-associated macrophages (TAMs) from the immunosuppressive M2 phenotype to the pro-inflammatory M1 phenotype, increases chemokine production, recruits T and NK cells, and downregulates TGF-β and T_reg_-mediated immunosuppression [3, 24]. LDRT can synergize with immunotherapies at various stages, amplifying their efficacy (Fig. 3). By optimizing the TME and immune cell recruitment, this combination strategy holds promise for improving outcomes in metastatic cancers.

Notably, LDRT-led polarization of M1 macrophages results in the secretion of cytokines/chemokines such as IL-12, IFN-γ, and RANTES, which recruit effector T cells and promote normalization of the tumor vasculature. Our recent findings build upon Klug et al.‘s findings, reinforcing that LDRT promotes a phenotypic shift of tumor-associated macrophages (TAMs) from the immunosuppressive M2 state to the antitumor M1 phenotype [15]. This macrophage polarization facilitates the recruitment of CD4 + T cells and NK cells while simultaneously downregulating TGF-β, a key inhibitory cytokine [3]. Expanding on this, we conducted proteomic analyses to examine the TME-specific changes induced by LDRT, revealing elevated levels of Granzyme B, MIP1α, and CD137 (4-1BB) within tumor-infiltrating CD4 + T cells, signifying enhanced activation and effector functionality. These findings underscore the multifaceted role of LDRT in modifying the tumor immune microenvironment [29]. Further support for these insights was identified in a randomized phase II trial evaluating the combination of PD-L1 and CTLA-4 inhibitors with either LDRT (0.5 Gy per fraction) or hypofractionated radiation (HFRT, 3 fractions of 24 Gy) in patients with metastatic colorectal cancer. Both radiation regimens were found to modulate local immune responses and systemic immunogenicity, highlighting the potential of LDRT in synergizing with checkpoint inhibitors [30]. Moreover, preclinical studies demonstrated that applying LDRT to murine tumors enhances T-cell infiltration and amplifies the response to combination immunotherapy driven by interferon-dependent mechanisms. Together, these findings illustrate the profound immunomodulatory effects of LDRT, emphasizing its value as a complementary approach to existing cancer therapies [17].

The potential of LDRT has gained considerable attention in treating tumors that are classified as immune-cold or immune-desert environments, such as liver metastasis or uveal melanoma [21]. Recent studies have highlighted the promising combination of LDRT with ICI, a strategy to overcome the immunosuppressive tumor microenvironment (TME) and improve therapeutic outcomes [3]. LDRT not only alters the physical characteristics of the tumor by reducing intratumoral pressure, but it also facilitates better drug delivery and retention within the tumor, increasing the effectiveness of subsequent therapies [31]. Further enhancing this strategy, the Radscopal Method, which involves delivering LDRT to secondary metastatic sites while applying high-dose radiation therapy (HDRT) to the primary tumor, significantly amplifies the systemic antitumor immune response [24]. This combination approach has improved treatment outcomes by expanding the therapeutic scope of radiation and immunotherapy. Additionally, LDRT, in conjunction with low-dose targeted radionuclide therapy, sensitizes previously immunologically cold tumors to ICI, resulting in a previously unattainable level of responsiveness [3, 24, 32, 33]. Together, these findings underscore the importance of LDRT in reshaping the TME to enhance local tumor control and drive systemic antitumor immunity. The combination of LDRT with immunotherapies presents a promising avenue for improving the treatment of difficult-to-target, immune-resistant tumors [34]. Figure 3 illustrates how immunotherapies interact with the modulated TME to boost immune responses.

## Combination of LDRT and immunotherapies in metastatic melanoma

LDRT has demonstrated significant potential to alter the tumor microenvironment and enhance immune responses. As a result, extensive preclinical and clinical studies have highlighted the benefits of combining LDRT with immunotherapy. The integration of LDRT with treatments like checkpoint inhibitors and cell therapy offers substantial promise in cancer management, primarily due to LDRT’s capacity to strengthen the function of effector cells. Table 1 lists clinical and preclinical studies utilizing LDRT and immunotherapy.

### LDRT with checkpoint inhibitors(s)

Immune checkpoint inhibition has significantly improved overall survival rates among melanoma patients. Nonetheless, challenges arise in cases of tumor recurrence following therapy, instances of resistance, and particularly in patients with immunologically cold uveal melanoma. Liver metastases exacerbate the challenge by significantly diminishing immunotherapeutic efficacy, principally due to the minimal immune response elicited in such cases [42]. Remarkably, a stage-IV melanoma patient demonstrated a complete response following LDRT to liver metastases, having undergone pretreatment with T-cell therapy [21]. Preclinical studies utilizing mouse models of melanoma and other cancers have revealed significant tumor control when combining LDRT with ICI, surpassing the efficacy of either treatment alone or in one such study using a B16 melanoma mouse model, LDRT treatment (50 cGy administered twice daily to a total dose of 500 cGy) notably enhanced the immunological response to PDL1 blockade. This dual therapy approach substantially decreased tumor growth, improved overall survival, and led to a remarkable 40% increase in complete response rate [38]. Furthermore, in a preclinical study involving an immunologically cold cutaneous melanoma B78 cell model derived from murine skin cancer B16 cells, flank tumors showed elimination when treated with an ISV regimen of radiation (12 Gy × 1 F), immune-cytokine, and anti-CTLA-4. However, this regimen had minimal impact on intracranial tumors. Interestingly, mice subjected to a precisely timed low-dose whole-brain radiation treatment (WBRT, 4 Gy × 1 F) demonstrated significantly enhanced survival rates and improved control over metastatic tumors [22]. This indicates that LDRT can sensitize an otherwise immunologically resistant tumor to ICB therapy. Low-dose targeted radionuclide therapy (TRT), specifically with 90Y-NM600, has been shown to alter the response of ICB in immunologically cold syngeneic B78 melanoma tumors. Notably, low-dose TRT has been reported to enhance NK cell activity significantly and increase tumor-infiltrating myeloid cells, resulting in an elevated ratio of CD8+ to suppressor T regulatory cells [34]. Neither checkpoint blockade nor LDRT alone significantly impacted melanoma tumor growth. However, combining both treatments led to a dramatic reduction in tumor growth rate, prolonged survival, and a 40% complete response rate in mice receiving dual therapy [17]. Notably, PD-L1 blockade alone enhanced the proliferation, antigen experience, activation, and cytotoxicity of circulating and tumor-resident CD8+ T-cells. In contrast, LDRT alone increased the prevalence of antigen-experienced circulating CD8+ T-cells and activated tumor-resident CD8+ T-cells but had no significant effect on their proliferation in either compartment [38]. These findings demonstrate that while neither LDRT nor checkpoint blockade is sufficient as a standalone treatment, their combination holds significant potential to sensitize immunologically resistant tumors to immunotherapy, offering a promising strategy for clinical application.

**Table 1:** Highlighted clinical and preclinical studies utilizing LDRT and immunotherapy

Model	Treatment	Cancer	Radiation dose	Effects	Reference
Preclinical	LD-TBI and IL-2 Synergism	Melanoma with lung metastasis (B16F1 malignant melanoma in mice)	0.75 Gy/f	Significant increase in NK cells and macrophages infiltrating the metastatic site. CD122 + expression and NK and T cells in peripheral blood and spleen were significantly increased.	[35]
Preclinical	LD-TBI and IL-2	Melanoma (B16F1) murine metastatic malignant melanoma model	0.75 Gy/f	The combination of high doses of IL-2 and LDWBI (either dose) resulted in a significant reduction in tumor load and an increase in tumor invasion of NK cells.	[36]
Preclinical	Localized radiotherapy + LDWBI + gene therapy by intratumor injection of Egr-mIL-18- B7.1	Melanoma (B16)	0.075 Gy	Improved survival rate, reduced tumor weight and lung metastasis, inhibition of tumor capillaries. Enhanced activity of NK cells and cytotoxic T lymphocyte (CTL), and increased secretion of interferon-γ (IFN-γ).	[37]
Clinical stage-IV melanoma	LDRT + PD1	Liver metastasis in Advanced melanoma	1.4 Gy/4f (5.6 Gy)	Complete response was observed in a, receiving LDRT to liver metastases. The patient was pretreated with ICB and T-cell therapy.	[21]
Preclinical	LDRT +PD-L1	B16 melanoma mouse model	500 cGy	Significantly reduced tumor growth rate, improved survival, and an increase of 40% complete response rate.	[38]
Preclinical	Abscopal +IC+ αCTLA4 + WBRT	Melanoma mouse model	Whole brain LDRT (WBRT) 4 Gy × 1f	Significantly improved survival and cranial metastatic tumor control	[22]
Preclinical	TRT+ ICB	Immunologically cold syngeneic B78 melanoma tumors	Low dose of targeted radionuclide therapy (TRT)	Improved NK cells, tumor infiltrating myeloid cells along with increase in the ratio of CD8+ to suppressor T regulatory cells	[34]
Preclinical clinical	Oncolytic Virus + LDRT + α-PD1	Melanoma (B16F1) mouse model	6 Gy	Reduced tumor growth and prolonged survival via conversion of ‘cold’ tumors to ‘hot’, via a CD8+ T cell-dependent and IL-1α-dependent mechanism and increased PD-1/PD-L1 expression.	[39]
Clinical	Oncolytic Virus +LDRT +ICB	PD-1-refractory patient with cutaneous squamous cell carcinoma	2.5 Gy/1f for 20 fractions	Prolonged control and survival.	[39]
Preclinical	vaccinia virus to express NIS before RT ^131^I treatment	Prostate Carcinoma Cells	-	Markedly improved the accumulation of radioiodine mediated by intratumoral NIS protein expression, and the combination suppressed prostate carcinoma cells compared to either OVs alone or ^131^I alone	[40]
Preclinical	^131^I-iodide + vaccinia virus (GLV-1h153)	orthotopic triple-negative breast cancer (TNBC) murine model	-	Resulted in a 6-fold increase in tumor regression as compared to the virus-only treatment group.	[41]

## LDRT combination with CAR-T cell infusion

The emergence of chimeric antigen receptor (CAR) engineering has propelled the advancement of potent cellular therapies for cancer. Referred to as “living drugs,” these modified immune effector cells have entered clinical practice. While CAR-T cell-based treatments have shown significant clinical success, logistical challenges and associated toxicities remain recognized limitations [5]. These engineered immune cells hold therapeutic promise for cancer-related conditions and inflammatory and infectious diseases [43]. CAR-T cell therapies have transformed the management of hematological malignancies like leukemia and lymphoma. However, effectively treating solid tumors with CAR-T cells remains a significant challenge in the field, with attempts to surmount these obstacles yielding limited success thus far. Numerous preclinical studies in melanoma models have explored CAR T cell approaches targeting various tumor antigens including CD16 [44], HER2 [45], GP100-HLA-A2 complex [46], CD126 [47], VEGFR-2 [48], CD20 & MCSP [49], MCSP [50], gp100 and MCSP [51], GD2 [52], GD3 [53], B7-H3 [54], and CD70 [55]. Likewise, several clinical trials have been undertaken in melanoma, employing CAR-T cells directed against various target tumor antigens, including VEGFR2 [56], GD2 in uveal melanoma [57], cMet [58], hCD70 [59], gp100 [60], NY-ESO-1 [61] IL13Ralpha2 [62], B7H3, and Bispecific B7H3xCD19 [63].

**Fig. 4:**
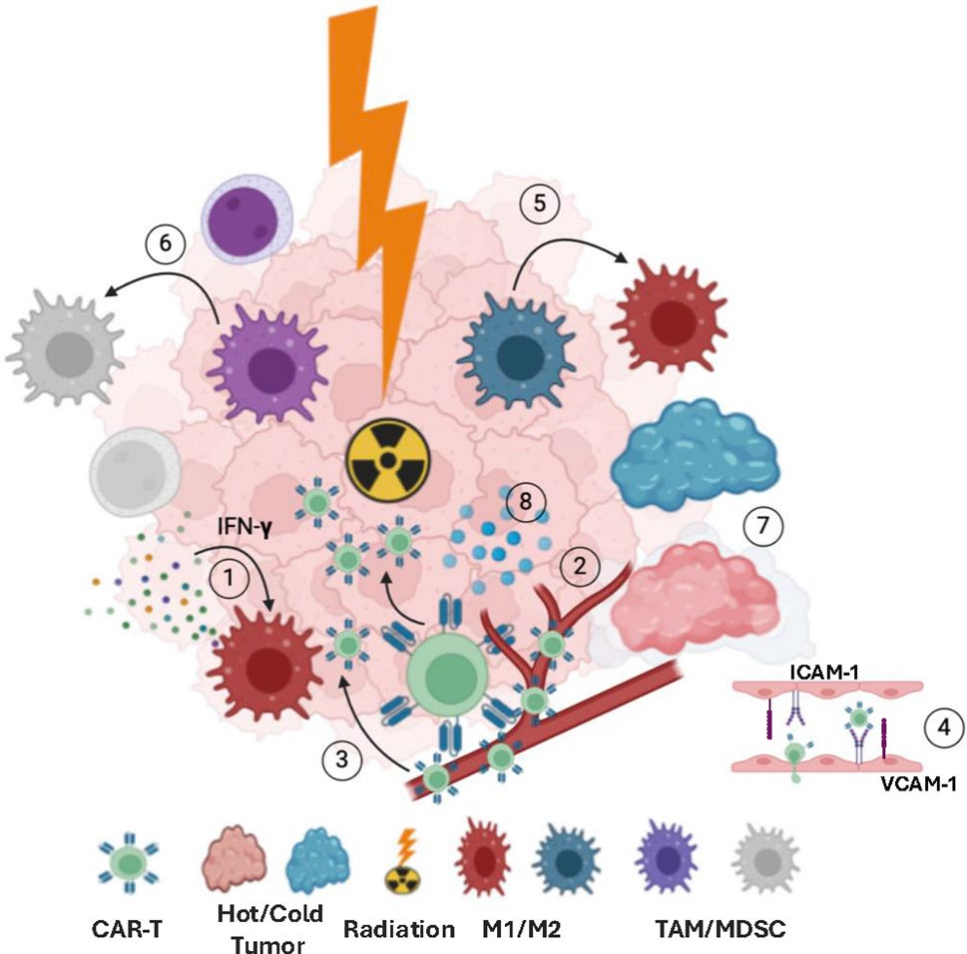
Radiotherapy amplifies the effectiveness of CAR-T cell therapy in combination treatments. (1,2) Radiation-induced IFN-γ triggers the secretion of chemokines such as CXCL9, CXCL10, and CXCL11, which help guide CAR-T cells to the tumor site. (3) Reducing barriers within the tumor microenvironment (TME), facilitates CAR-T cell infiltration. (4) Radiation also increases the expression of ICAM-1 and VCAM-1 on tumor blood vessels, further aiding CAR-T cell infiltration. (5) Radiotherapy shifts macrophages in the TME from the M2 to the M1 phenotype. (6) Additionally, radiation decreases the presence of tumor-associated macrophages (TAM) and myeloid-derived suppressor cells (MDSC). (7) By increasing the expression of proinflammatory cytokines, radiation transforms the TME from an immunosuppressive “cold” state to a more immune-active “hot” state. (8) This enhances the function of infiltrating CAR-T cells and supports the expansion of CAR-T cells. This figure was generated using BioRender.com

We recently reported a compelling finding that LDRT enhances CAR-T cell infiltration into the solid tumor microenvironment, leading to improved tumor control and extended survival. This approach presents a promising strategy to address the challenges associated with CAR-T therapy in solid tumors [64]. While high-dose radiotherapy leads to cellular detrimental effects, including apoptosis, low-dose radiation (LDR) has shown promise in benefiting patients by stimulating an anti-tumor immune response and addressing antigen escape [65–67]. Reports emphasize the synergistic potential of RT and CAR-T cell therapy in controlling tumor growth [68]. In a leukemia model, LDRT alters the tumor-intrinsic transcriptional state in a computable “Death Receptor Score,” which reflects a tumor’s innate sensitivity to CAR-T cells. This Death Receptor Score transiently increases with low-dose total-tumor irradiation (TTI), and independently correlates with outcomes in banked samples from a series of patients with ALL [68]. Furthermore, Weiss et al. showed that a subtherapeutic dose of local RT in combination with CAR-T cells targeting NKG2D showed synergy against mouse glioma by promoting CAR-T cell migration and enhancing their cytolytic function [69]. As illustrated in Fig. 3, multiple tumor models demonstrate that RT induces the release of chemokines such as CXCL9, CXCL10, and CXCL11, which enhance T-cell trafficking. Moreover, RT upregulates adhesion molecules like intracellular adhesion molecule 1 (ICAM-1) and vascular cell adhesion molecule 1 (VCAM-1) on tumor blood vessels, facilitating CAR-T cell infiltration into the tumor microenvironment. RT also reshapes the immune landscape within the tumor microenvironment (TME) by altering immune cell composition and increasing the expression of immune-stimulatory cytokines, further enhancing the functional activity of effector T cells. RT-induced release of the chemokine IL-8 was co-opted to drive the migration, antitumor efficacy, and persistence of CAR-T cells modified to express the cognate IL-8 receptors CXCR1 and CXCR2 in murine models of human glioma, breast, and pancreatic cancer [70]. Taken together, these effects render a more favorable TME that amplifies the efficacy of CAR-T therapy, as shown in Fig. 4.

Building on these findings, combining LDRT with CAR-T cell therapy offers significant potential for improving tumor response and modifying the TME to support immune cell activity. This combination strategy may also reduce treatment-related toxicities, including autologous graft-versus-host disease (GvHD), cytokine release syndrome, and Immune Effector Cell-Associated Neurotoxicity Syndrome (ICANS), while enhancing the overall effectiveness of CAR-T therapy. Such an approach could lower the required therapeutic doses, addressing challenges such as manufacturing time (typically 2–4 weeks for autologous CAR-T therapy) and high costs.

Despite these promising implications, no reports have explored the use of LDRT and CAR-T synergy in melanoma models. Our review highlights this critical research gap, encouraging melanoma researchers to investigate this novel combination in preclinical and clinical settings. By doing so, this area of research could unlock new therapeutic avenues and significantly improve outcomes for melanoma patients.

## LDRT combination with CAR-NK cell infusion

**Fig. 5:**
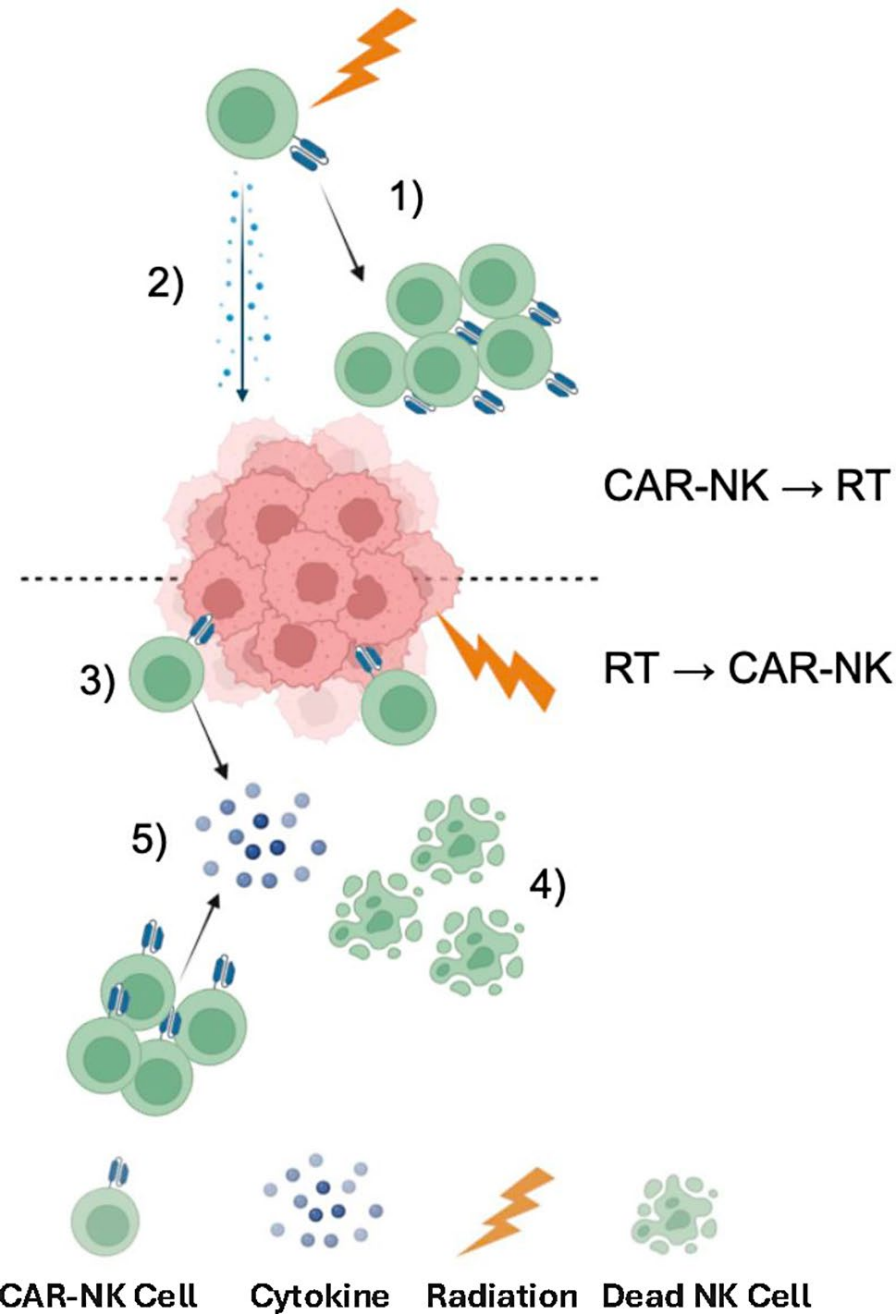
The impact of radiation therapy on NK and CAR-NK cell activity, infiltration, and antitumor cytolytic ability. Radiation therapy is key in enhancing the activity and infiltration of NK and CAR-NK cells within the tumor microenvironment. The timing of radiotherapy mediates several crucial effects: (1) Radiation promotes the expansion of natural killer (NK) cells, boosting their cytotoxic potential. (2) It activates NK cells, stimulating the secretion of effector cytokines like IFN-γ and TNF-α, strengthening their antitumor responses. (3) Radiotherapy reduces tumor burden, facilitating the infiltration of CAR-NK cells into the tumor site. (4) Radiation-induced lymphopenia creates additional space for CAR-NK cell expansion, enhancing their therapeutic efficacy. (5) Radiation also induces the release of chemokines, which attract CAR-NK cells to the tumor, optimizing their localization and activity. The synergy between radiation therapy and CAR-NK cell therapy underscores the potential of this combined approach for cancer treatment

There is increasing interest in exploring alternative immune effector cell types for CAR therapy. Among these, natural killer (NK) cells stand out as a promising choice due to their innate cytotoxic capability and independence from antigen presentation in the MHC pathway. NK cells are integral to the innate immune system and can directly target and kill tumors, contributing to immunosurveillance and antitumor immune responses. Their innate cytotoxic potential against malignancies can be augmented by incorporating CAR technology into NK cells. As observed in Fig. 5, radiation therapy enhances the activity, infiltration, and antitumor efficacy of NK cells within the tumor microenvironment. RT promotes NK cell expansion and activation, increasing cytotoxic potential and cytokine secretion (e.g., IFN-γ and TNF-α) [71]. Ionizing radiation therapy (RT) induces the secretion of chemokines, notably interleukin-8 (IL-8). In ex vivo human pancreatic cancer specimens, RT-induced IL-8 effectively recruits unmodified natural killer (NK) cells, demonstrating a synergistic potential for combining radiation therapy with NK cell-based treatments to enhance cancer therapy outcomes [72]. NK cell therapy provides a significant advantage by functioning independently of major histocompatibility complex (MHC) antigen presentation, positioning it as a promising allogeneic treatment option. Unlike allogeneic CAR-T therapy, which is associated with graft-versus-host disease (GVHD), CAR-NK therapy exhibits minimal toxicity and eliminates the risk of GVHD. This feature eliminates the requirement for human leukocyte antigen (HLA) matching, positioning CAR-NK therapy as a safer and more accessible option for immunotherapy [73, 74].

So far, LDRT has shown positive effects on NK cells in melanoma tumor microenvironment. For example, radiofrequency radiation (RFR) has been found to reprogram the TIME into an antitumor phenotype in pulmonary metastatic melanoma, promoting the active transformation of tumor-infiltrating CD8+ T and NK cells [75]. Furthermore, low-dose radiation (75 to 150 mGy) has significantly enhanced the expansion and secretion of effector proteins such as IFN-γ and TNF-α from NK cells [76, 77]. Similarly, studies involving tumor-bearing rats subjected to low-dose total-body irradiation (0.1–0.2 Gy) reported significantly reduced tumor metastases and increased NK cell cytolytic activity [78, 79]. Despite these findings, no research currently examines the combination of LDRT and CAR-NK therapy in melanoma models. This represents a notable scientific gap, and our review encourages melanoma researchers to explore this promising combination in appropriate preclinical models.

## LDRT in combination with cytokines

**Fig. 6:**
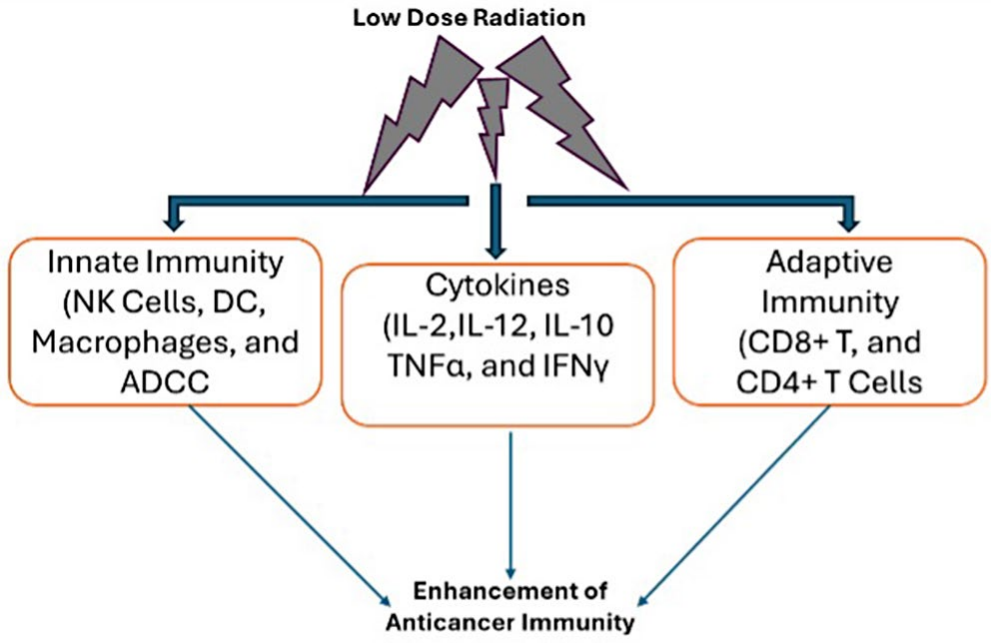
Combining various cytokines with low-dose radiotherapy (LDRT) demonstrates significant direct antitumor potential. LDRT enhances the immune-modulating effects of cytokines by amplifying their ability to stimulate the immune system. This approach directly boosts innate immunity and promotes the production of antitumor proinflammatory cytokines, strengthening the overall antitumor response

The synergistic effects of RT and cytokine treatment have gained increasing attention for their ability to enhance the antitumor immune response. Combining LDRT with cytokine therapy significantly enhances antitumor responses [80, 81]. LDRT amplifies the immune-stimulating effects of cytokines, boosting innate immunity and promoting the production of proinflammatory cytokines, thereby strengthening the overall antitumor immune response (Fig. 6). RT serves as a primer for the tumor microenvironment, preparing it for subsequent immunotherapies, including cytokine therapy [82]. Preclinical and clinical studies have demonstrated the potential of combining RT with various cytokines, such as IL-12 and IL-2, to amplify antitumor immune responses [83–85].

A phase 2 clinical trial by Bulgareli and Piccinini (2021) evaluated the combination of RT and IL-2 in patients with metastatic melanoma and renal cell carcinoma. Patients received three daily doses of 6–12 Gy of radiation alongside IL-2, administered via continuous infusion over 72 h and repeated every three weeks for a total of 4 cycles. The study found that 15.7% of patients had a partial response, and 36.8% exhibited stable disease, resulting in a disease control rate of 52.6%. These findings suggest that combination therapy could be a promising option for patients with advanced melanoma, especially those undergoing standard treatments [2]. Recent advances have also explored the delivery of immunocytokines [86], such as NHS-IL2, which combines the third-generation tumor necrosis therapy antibody NHS76 with IL-2. This engineered cytokine has shown promise in selectively activating high-affinity IL-2 receptors. In animal models, Heuvel et al. demonstrated that combining NHS-IL2 with low-dose RT and cisplatin led to significant tumor regression (80-100%). In a phase-IB clinical trial for metastatic non-small cell lung carcinoma patients, the combination of local irradiation (4 Gy x 5) and escalating doses of NHS-IL2 (0.15 mg/kg, 0.30 mg/kg, and 0.45 mg/kg) administered intravenously over three consecutive days every three weeks resulted in long-term survival in two patients, one of whom achieved long-term tumor control. Although adverse events like fatigue, anorexia, rash, and thyroid dysfunction were reported, these results support the efficacy of combining low-dose radiation with NHS-IL2 in enhancing antitumor responses [83]. In a murine melanoma model, an in-situ vaccination (ISV) regimen combining RT, intratumoral injection of immune-cytokine (anti-GD2 antibody fused to IL-2), and the ICI anti-CTLA-4 demonstrated strong efficacy in eliminating peripheral flank tumors. However, its effects on intracranial tumors were more modest [85].

IL-12 is critical for Th1 differentiation, CD4 + T cell reactivation, CD8+ T cell and NK cell cytotoxicity, and survival [87]. However, its clinical application is limited by dose-limiting toxicities, with a maximum tolerated dose of rhIL-12 reported as 500 ng/kg in a phase I trial [88]. A fusion of IL-12 (NHS-IL12), heterodimers with DNA/histone-binding antibodies (NHS76), targets neoplastic lesions by binding DNA fragments in histones [89]. In mouse models, NHS-IL12 shows superior antitumor effects compared to recombinant IL-12, mainly via CD8+ T cells. Its efficacy is enhanced with tumor vaccines, radiotherapy, or chemotherapy [90]. While a phase I trial confirmed its safety at doses up to 16.8 µg/kg, no significant antitumor effects were observed in patients [91]. Developing genetically engineered cytokines holds significant potential for advancing antitumor therapies, particularly for aggressive cancers like metastatic melanoma.

These findings underscore the promise of combining low-dose radiotherapy with immunotherapy, highlighting the potential for improved outcomes in treating advanced melanoma and other cancers.

## Tumor infiltrating lymphocyte therapy combination with LDRT

**Fig. 7:**
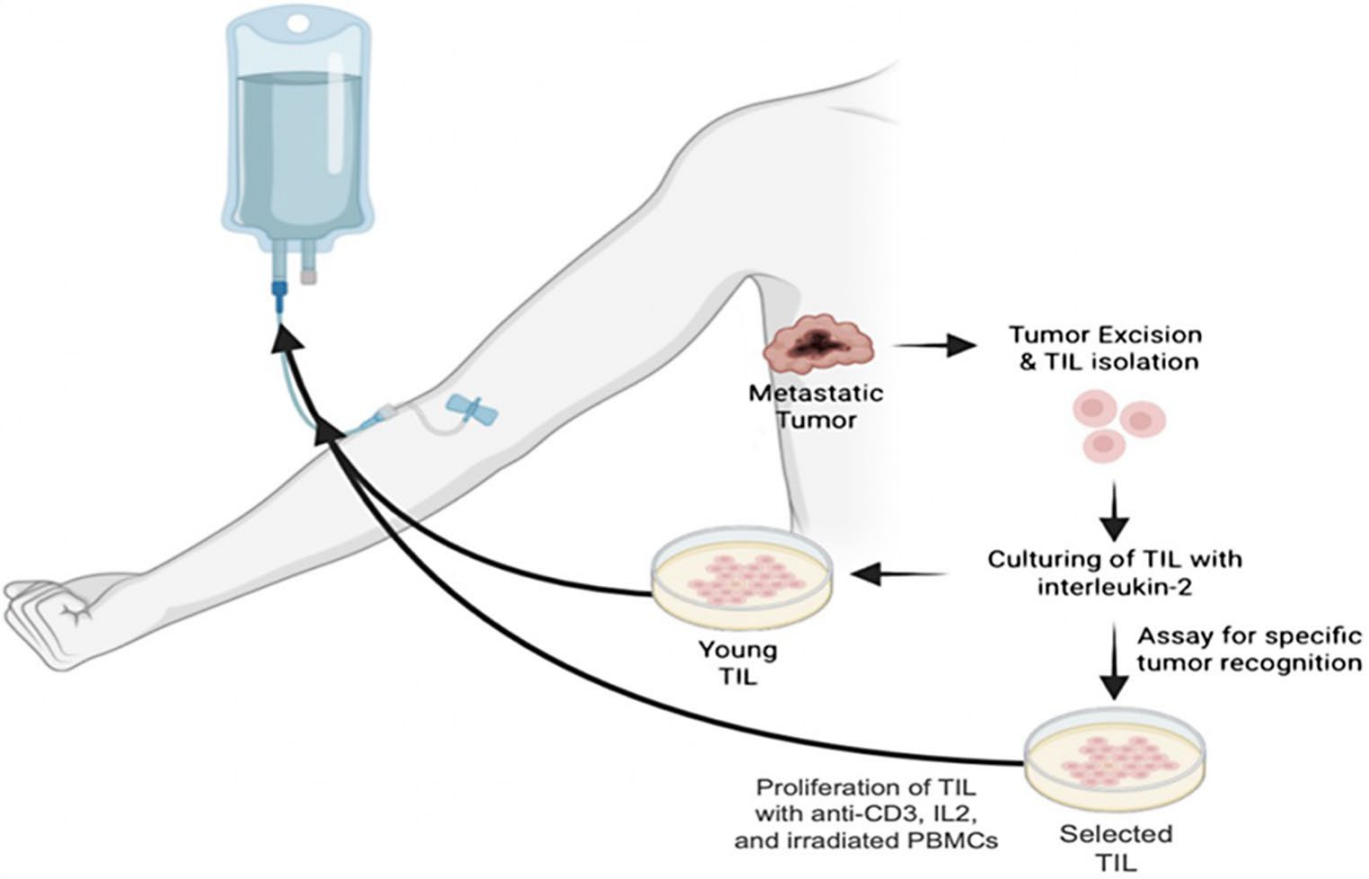
Schematic representation of TILs isolation from the metastatic lesions and development into a therapeutic product followed by reinfusion

In a groundbreaking development, the FDA recently approved the first-ever tumor-infiltrating lymphocyte (TIL) therapy, Iovance’s *lifileucel*, for advanced melanoma. This approval represents a pivotal moment in cancer treatment, signaling a transformative step forward in managing solid tumors. TIL therapy, once overshadowed by approaches such as ICI and chimeric antigen receptor (CAR) T-cell therapies, is experiencing a renaissance. Figure 7 shows the isolation of TILs from a metastatic lesion followed by their expansion. With renewed focus and proven efficacy, TIL therapy’s relevance in treating advanced melanoma is gaining momentum, meriting in-depth exploration of its potential [92]. Adoptive cell therapy using TILs has demonstrated remarkable promise in metastatic melanoma. Clinical responses have been observed in checkpoint inhibitor-naïve and -refractory cutaneous melanoma cases. Notably, uveal melanoma (UM)—a subtype historically resistant to ICI—has shown encouraging clinical results with TIL therapy in early studies. For instance, a phase II trial of *lifileucel* in advanced melanoma patients who had previously undergone ICI and targeted therapy (BRAF, MEK inhibitors) reported durable and significant responses, reaffirming the potential of TILs even in heavily pretreated individuals [93]. These trials underscore *lifileucel’s* potential as a one-time treatment for patients with limited therapeutic options, particularly in cases of ICI-refractory disease [94].

Despite the advancements brought by ICIs and targeted therapies, nearly half of patients with advanced melanoma fail to achieve durable responses. This gap has propelled interest in TIL therapy as an alternative. Phase I/II trials of TILs have shown notable clinical efficacy, with phase III data revealing superior progression-free survival (25.8 months) compared to ipilimumab-treated patients (18.9 months) [95]. In contrast to CAR T-cell therapies, which face significant barriers in solid tumors, TILs appear uniquely suited for such malignancies. A phase III randomized clinical trial for metastatic melanoma further highlighted the potential of TIL therapy, with findings from the Netherlands Cancer Institute demonstrating a 50% response rate among treated patients and durable complete responses. These findings also underscored the critical role of neoantigen-specific T-cell reactivity, laying the groundwork for larger multicenter trials [96].

TIL therapy’s efficacy has also been explored in uveal melanoma, a historically challenging subtype to treat. A phase II trial targeting metastatic uveal melanoma achieved objective tumor regression in 35% of patients, with one complete response and six partial responses [17]. Notably, some responders had prior ICI-refractory disease, highlighting TIL therapy’s potential even in heavily pretreated populations [97]. Parallel innovations at MD Anderson Cancer Center have optimized TIL harvesting and expansion using a three-signal culture method involving TCR, 4-1BB stimulation, and high-dose IL-2. This strategy achieved a disease control rate of 66% (22% partial response and 44% stable disease), further advancing TIL therapy’s efficacy [98].

Beyond ex vivo innovations, the tumor microenvironment itself has been manipulated to improve TIL therapy outcomes. Radiation therapy (RT), chemotherapy (CT), and combined chemo-radiation therapy (CRT) have been shown to increase CD3 + and CD8+ TIL density in treated tumors significantly. High pretreatment TIL levels have been positively associated with prolonged disease-free survival (DFS) and overall survival, highlighting the synergistic effects of these modalities [4].

Recent innovations in LDRT have provided additional insights into how TIL therapy can be enhanced. LDRT has improved ex vivo TIL expansion while increasing the antitumor activity of adoptively transferred T cells. Combining TIL therapy with radiation represents a promising strategy to boost response rates, particularly in recurrent or metastatic melanoma [99].

The FDA approval of *lifileucel* marks a new era in TIL therapy, validating its potential as a viable treatment for advanced melanoma. TIL therapy’s resurgence, supported by clinical trial data and innovations in combination therapies, highlights its role in filling critical gaps left by ICIs and CAR T-cell therapies. The integration of TIL therapy with radiation or other adjuncts not only offers a means to enhance efficacy but also provides a pathway to address the unmet needs of refractory and metastatic disease. As ongoing studies refine this approach, TIL therapy may become a cornerstone of immuno-oncology, offering renewed hope for patients battling advanced melanoma.

## Combining oncolytic virus therapy with LDRT

**Fig. 8:**
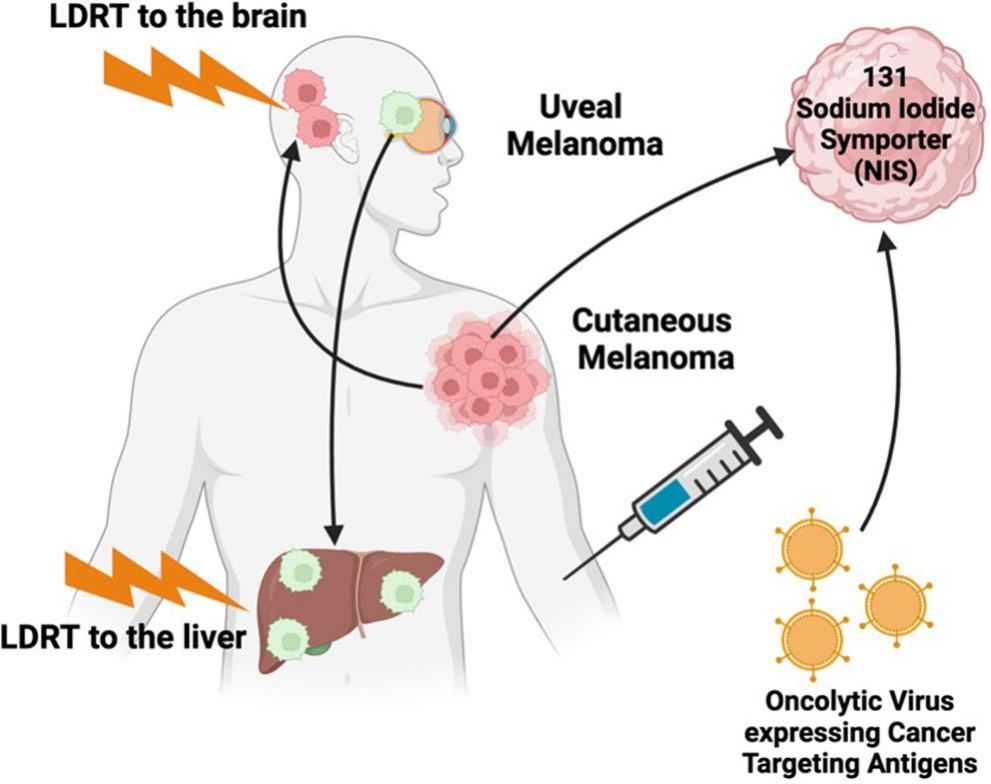
Oncolytic virus-mediated immunotherapy combined with low-dose radiation targeted to secondary metastatic sites presents a novel treatment strategy for advanced uveal melanoma (particularly affecting the liver), as well as cutaneous melanoma (particularly affecting the brain)

Oncolytic viruses (OVs) represent an innovative therapeutic avenue for targeting metastatic sites in advanced melanoma, such as brain metastases in cutaneous melanoma and liver metastases in uveal melanoma. These engineered viruses selectively infect and lyse tumor cells while simultaneously eliciting immune-mediated tumor regression [6]. *Talimogene laherparepvec* (T-VEC), the first FDA-approved oncolytic virus, exemplifies this approach. T-VEC is a modified herpes simplex virus engineered to express granulocyte-macrophage colony-stimulating factor (GM-CSF), enhancing immune cell recruitment and tumor destruction. Its approval for unresectable cutaneous melanoma is based on improved response rates compared to GM-CSF alone [100]. Figure 8 illustrates how combining OVs and LDRT may amplify these effects, offering a promising therapeutic synergy.

Numerous oncolytic viruses are under investigation in solid tumors, including melanoma, employing diverse viral vectors and transgenes. Adenovirus 5 and herpes simplex virus (HSV)-based OVs are commonly administered intratumorally and are often engineered with GM-CSF to recruit dendritic cells (DCs) and NK cells [101, 102]. In a phase I clinical trial, the vesicular stomatitis virus (VSV) expressing interferon beta (IFN-β) and tyrosinase-related protein 1 (TYRP1) demonstrated safety and induced dose-dependent immunogenicity and T-cell responses in uveal melanoma [6]. Similarly, the enteric cytopathic human orphan virus type 7 (ECHO-7), also known as Rigvir, exhibited preclinical cytolytic activity against human uveal melanoma cells [103]. A phase 1 trial investigated the intravenous administration of oncolytic adenovirus ICOVIR-5 in patients with cutaneous and uveal melanoma [104]. These findings highlight the versatility and therapeutic potential of OVs in melanoma treatment.

Preclinical studies further support the efficacy of combining OVs with radiotherapy. For example, in an anti-PD-1 refractory melanoma mouse model, combining oncolytic virus particles (OncoVECmGMCSF/mT-VEC) with radiation therapy (6 Gy / 1 fraction) significantly reduced tumor growth and improved survival. This strategy transformed immunologically “cold” tumors into “hot” ones, relying on CD8+ T cell-dependent and IL-1α-dependent mechanisms. A notable clinical case involved a PD-1-refractory patient with cutaneous squamous cell carcinoma who achieved prolonged disease control with a combination of OV, radiation therapy, and ICI [39]. Such evidence underscores the transformative potential of OVs in enhancing immune responses and overcoming resistance mechanisms.

Oncolytic viruses are also being engineered to disrupt DNA damage repair (DDR), rendering tumors more susceptible to radiation therapy. For instance, adenovirus E4orf6 protein inhibits DNA repair pathways, increasing tumor radiosensitivity [105]. The vaccinia virus GLV-1h153 has shown promise in triple-negative breast cancer (TNBC) by increasing intratumoral radionuclide accumulation and enhancing radiation therapy efficacy [41]. Other genetically modified OVs express proteins, such as the sodium iodide symporter (NIS), which selectively facilitates radionuclide uptake, improving radiotherapy precision and safety [106–108] (Fig. 8). For example, a vaccinia virus expressing NIS combined with ^131^I radiation therapy significantly suppressed prostate carcinoma growth compared to either treatment alone [40]. These innovative approaches illustrate the versatility of OVs in improving radiotherapy outcomes. An oncolytic NDV expressing an anti-CTLA4 antibody acted as a radio-enhancing agent, synergizing with standard radiation to enhance tumor repression [109].

Despite advancements in immune checkpoint inhibitors, metastatic uveal melanoma (mUM) continues to exhibit limited responses due to its low tumor mutational burden (TMB) and reduced PD-L1 expression, which impairs neoantigen recognition by tumor-specific T cells [110–112]. Tebentafusp, an immune-mobilizing monoclonal T-cell receptor (TCR) therapeutic, represents a significant breakthrough for mUM, targeting HLA-*A*0201 and gp100 antigens with a median overall survival of 15.3 months [113]. However, many mUM patients are HLA-A*0201* negative and ineligible for this therapy, underscoring the urgent need for alternative treatments. Oncolytic viruses present a promising immunotherapeutic option for these patients, offering a novel mechanism to stimulate anti-tumor immunity and potentially overcome resistance to existing therapies. Integrating oncolytic viruses with radiation therapy and immunotherapy has the potential to revolutionize melanoma treatment. These strategies aim to address the unmet needs of metastatic uveal melanoma patients, providing hope for improved outcomes through innovative and combinatorial approaches.

## Combination of Tumor Treating fields (TTFs) with LDRT

**Fig. 9:**
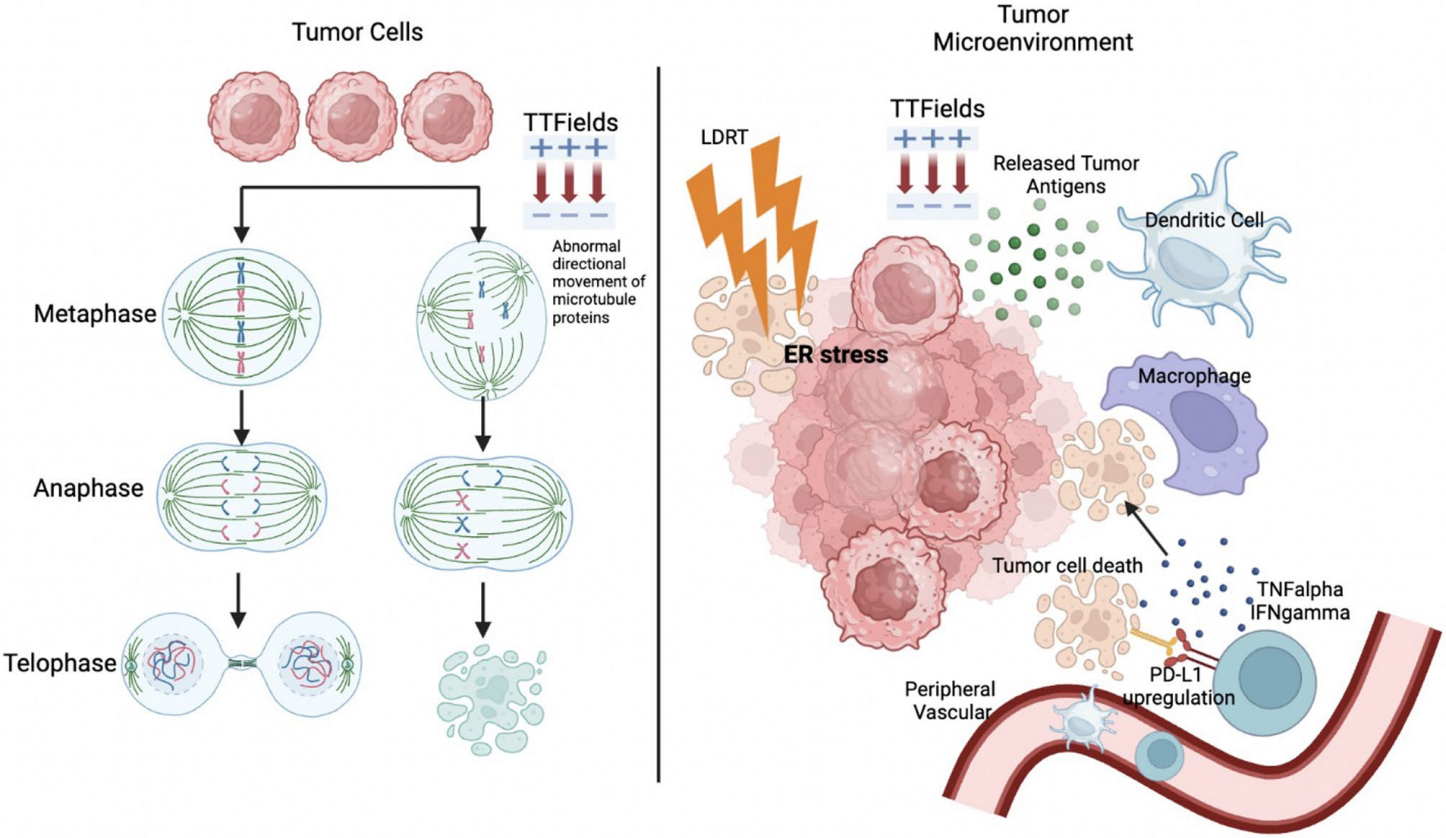
Tumor Treating Fields (TTFs) disrupt tumor mitosis and engage other mechanisms, including a potent synergistic antitumor immune response when combined with radiation therapy. The combination of TTFs and low-dose radiation therapy (LDRT) holds significant promise but requires detailed investigation. This area of research offers considerable potential for further exploration and development

Tumor Treating Fields (TTFields) represent a promising loco-regional treatment approach that utilizes low-intensity alternating electric fields (1–3 V/cm) with frequencies ranging from 100 to 400 kHz. These electric fields are delivered transdermally to tumors using two transducer arrays activated sequentially every second. This activation induces directional shifts in the electric field targeting the tumor. TTFields provide a non-invasive cancer therapy by disrupting cellular structures in metastatic tumors. The optimal alternating current (AC) frequency is tumor-specific, with glioblastoma, for instance, showing maximum benefit at 200 kHz [114]. The directionality of the applied electric field influences the efficacy of TTFields. Initial hypotheses suggested that TTFields acted on polarizable intracellular structures to disrupt mitosis (Fig. 9). Subsequent studies have confirmed this, demonstrating that the treatment’s effectiveness depends on the direction and amplitude of the electric field [114, 115]. This suggests that the efficacy of TTFields, as observed by subcellular structures, including its amplitude of effect, may depend on the direction of the imposed electric field. Kirson and colleagues further hypothesized that periodically changing the field direction would enhance efficacy, validated for direction-change intervals of 1 and 2 Hz. The mechanism of action of TTFields includes inducing antimitotic cell cycle arrest, triggering autophagy and endoplasmic reticulum (ER) stress in cancer cells, and causing DNA damage and replication stress. Notably, TTFields also stimulate antitumor immune responses. Tumors treated with TTFields exhibit increased infiltration of immune cells, including CD45 + cells, CD4+, and CD8 + lymphocytes, creating an immunologically “hot” tumor microenvironment compared to untreated “cold” tumors [116, 117].

Studies have highlighted a remarkable synergy between TTFields and ionizing RT. For instance, TTFields combined with RT effectively suppress cell migration and invasion in glioblastoma multiforme (GBM) cells by inhibiting proteins such as MMP-9 and vimentin [118]. These findings underscore TTFields’ potential to enhance RT outcomes by inhibiting double-stranded DNA damage repair, inducing mitotic catastrophe, and reducing tumor cell survival (Fig. 9) [119]. Based on this synergy, Novocure conducts clinical trials using TTFields in metastatic uveal melanoma (mUM). One such trial (NCT05004025) evaluates the NovoTTF-2001 device in combination with ICI, nivolumab, and ipilimumab. As many uveal melanoma cases recur in the liver, array patches are strategically placed on the abdomen and worn for at least 18 h daily. TTFields have already demonstrated efficacy in brain tumors, including glioblastoma multiforme, with FDA approval at 200 kHz for newly diagnosed and recurrent GBM and 150 kHz for malignant pleural mesothelioma.

In glioblastoma, pilot studies assessing the concurrent use of TTFields with RT and temozolomide (TMZ) in newly diagnosed patients have demonstrated safety and feasibility [120]. This combination significantly enhances therapeutic outcomes, making TTFields one of the most innovative and advanced modalities to complement radiation therapy in metastatic melanoma.

## Exploring the potential integration of cancer vaccines and low-dose radiation therapy (LDRT) in metastatic melanoma

### Combining low-dose radiation therapy and cancer vaccines: a promising strategy

**Fig. 10:**
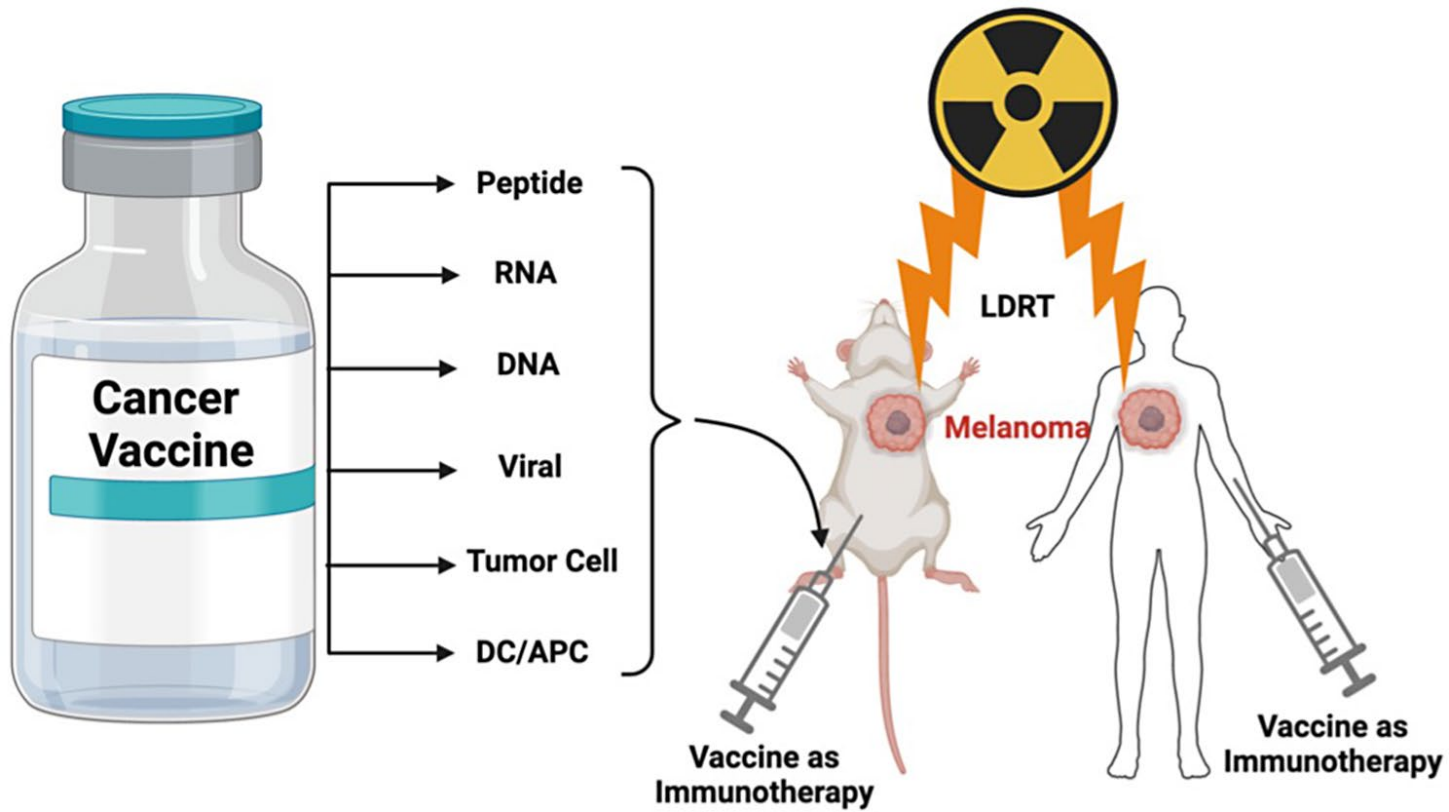
Combining low-dose radiation therapy (LDRT) with vaccines represents a winning strategy to enhance the antitumoral immune response, serving as a preventive, therapeutic, and personalized modality

Integrating radiation therapy, particularly LDRT, with cancer vaccines offers a promising new approach for managing metastatic solid tumors [22, 121–126]. Cancer vaccines, a form of immunotherapy, aim to educate the immune system to recognize and eliminate abnormal tumor cells. However, unlike vaccines for infectious diseases, the development of cancer vaccines is inherently complex. The challenges stem from the similarity between cancer and normal cells and the heterogeneity of tumor-specific antigens, which vary significantly between individuals [127, 128]. Cancer vaccines aim to stimulate anti-tumor immune responses by delivering tumor-associated antigens in various forms, including tumor cells, viruses, antigen-presenting cells (APCs) or dendritic cells, peptides, DNA, and RNA. Their primary goal is to overcome the immune-suppressive tumor environment and elicit robust cellular and humoral immune responses. These vaccines are classified into four main platforms based on their preparation methods: cell-based, virus-based, peptide-based, and nucleic acid-based vaccines [129, 130] (Fig. 10). Dendritic cell (DC) vaccines have shown significant efficacy in clinical trials. Nucleic acid vaccines, such as DNA and mRNA vaccines, have also gained traction due to their ability to encode tumor-associated antigens, initiate selective tumor responses, and generate robust immune activation [131–134].

### Radiation therapy as an in-situ vaccine

RT has been demonstrated to act as an in-situ vaccine by triggering systemic immune responses against localized tumors. This phenomenon, known as the abscopal effect, has been observed across various tumor types, including melanoma, non-small cell lung cancer, renal cell carcinoma, and hepatocellular carcinoma [110]. Through mechanisms such as enhanced antigen presentation and T-cell activation, RT destroys cancer cells locally and primes the immune system to target distant metastatic lesions [135, 136]. Indeed, mounting evidence supports the synergistic effects of RT and immunotherapies. These immune-modulating effects have made RT an attractive partner for cancer vaccines, with evidence suggesting that the combination significantly improves clinical outcomes, including disease-free survival and overall survival, across diverse cancer types [137–139]. Thus, radiation therapy’s ability to induce a tumor-specific immune response has led to its recognition as an in-situ vaccine.

### Enhancing cancer vaccines with Low-dose radiation therapy

The combination of LDRT and cancer vaccines is increasingly recognized for its potential to amplify tumor-specific immune responses (Fig. 10). For instance, in metastatic melanoma models, LDRT applied to the brain, combined with in-situ vaccines and anti-CTLA-4 therapy, transformed immunologically “cold” tumors into “hot” tumors, characterized by increased immune cell infiltration [22]. Similarly, combining mRNA-based vaccines with radiation therapy has demonstrated robust synergistic anti-tumor effects, including delayed tumor growth and complete tumor eradication in some cases. Gene expression analyses revealed increased antigen presentation, immune cell adhesion, innate immune system activation, significant downregulation of tumor-associated factors, and upregulation of tumor suppressors [140].

### Radiation priming of immune responses

Studies by Lugade et al. have highlighted the role of RT in priming immune responses. In murine melanoma models, RT increased antigen-presenting cells and IFN-γ-secreting T cells, enhancing tumor-specific CD8+ T-cell infiltration. Further investigation ha shown that RT can re-sensitize tumors resistant to anti-PD-1 therapy by increasing type I interferon secretion and MHC class I expression [141, 142]. A notable example includes a study combining RT with SD-101, a TLR9 agonist, which successfully converted “cold” tumors into T-cell-inflamed, “hot” tumors, thereby overcoming checkpoint blockade resistance [125, 143].

### LDRT and dendritic cell vaccines: a synergistic approach

Emerging evidence suggests that exposing dendritic cells (DCs) to LDRT enhances their immunogenic potential during vaccine preparation. In preclinical models, LDRT-exposed DCs demonstrated superior migratory capacity, increased T-cell proliferation induction, and heightened cytotoxic T lymphocyte (CTL) activity. Mice treated with DC vaccines exposed to LDRT exhibited improved survival, enhanced CTL infiltration into tumors, and increased tumor cell apoptosis. Upregulated serum levels of IFN-γ and interleukin-12 accompanied these effects [124]. Furthermore, LDRT has been shown to induce a hormetic effect, enhancing the activity of T cells and NK cells, and promoting the production of IL-12, a cytokine critical for Th1 differentiation and CTL activation [121–123, 144–147].

Moreover, a single exposure of DCs to LDRT at a dose of 0.2 Gy has been shown to significantly enhance the production of IL-12, a pivotal cytokine that drives the differentiation of naive T cells into Th1 cells and activates CD8 + CTLs. This increase in IL-12 production amplifies the DCs’ capacity to induce robust T-cell proliferation, thereby strengthening anti-tumor immune responses [148]. Additionally, another study demonstrated that exposure to LDRT at the same dose markedly improved the migratory ability of DCs, enabling them to reach lymphoid tissues and present antigens to T cells more effectively [149]. Together, these findings underscore the potential of integrating optimized doses of LDRT with advanced, specific cancer vaccines to enhance immune priming and activation. This combined approach holds great promise for overcoming the challenges associated with advanced melanoma, paving the way for more effective therapeutic strategies.

## Conclusions

LDRT has emerged as a transformative approach in treating metastatic melanoma, marking a significant advancement in oncology. Extensive research has highlighted LDRT’s unique ability to remodel the tumor microenvironment, fostering conditions that support a robust immune response. By stimulating the infiltration, activation, and functionality of immune cells essential for tumor destruction, LDRT enhances the efficacy of immunotherapeutic strategies. When integrated with immunotherapies—such as ICI, CAR-T cell therapy, cytokine therapy, TIL therapy, oncolytic virus therapy, and cancer vaccines—LDRT exhibits remarkable synergistic effects. These combinations improve tumor control and patient survival and address critical challenges, including tumor recurrence and resistance to conventional treatments. Additionally, LDRT facilitates the conversion of immunologically “cold” tumors into “hot” tumors, significantly enhancing their responsiveness to immunotherapy.

Overall, LDRT offers a compelling avenue for advancing metastatic melanoma treatment, providing new hope for improved outcomes and expanding therapeutic options for patients battling this aggressive disease. Its potential to reshape the standard of care underscores the importance of further research and clinical exploration to unlock its full capabilities.

### Future directions

The combination of LDRT and immunotherapy holds great promise in advancing the treatment landscape for metastatic melanoma. To fully harness its potential, several critical areas warrant further exploration. Optimizing treatment regimens is essential to maximize therapeutic efficacy while minimizing adverse effects. This optimization involves systematically investigating the sequencing, dosing schedules, and specific combinations of LDRT with various immunotherapeutic agents to determine the most effective protocols. Personalized treatment strategies are also paramount. Identifying reliable biomarkers to guide patient selection and tailor therapies to individual tumor profiles can significantly enhance treatment outcomes. Emerging combinations of LDRT with TTFs and oncolytic viruses represent exciting avenues. With their advanced antitumor mechanisms, these novel modalities demand comprehensive exploration to unlock their full therapeutic potential. Additionally, understanding and addressing mechanisms of treatment resistance remains a critical focus. Insights into resistance pathways can inform the development of innovative strategies to overcome these challenges and sustain durable responses. Beyond metastatic melanoma, LDRT’s applications are increasingly being considered for other cancers where immunotherapy has shown efficacy. Expanding research into integrating LDRT with targeted therapies, chemotherapy, and other novel approaches could reveal synergistic effects, broadening the spectrum of therapeutic possibilities. Integrating LDRT with immunotherapy marks a transformative shift in managing metastatic melanoma. However, realizing its full potential hinges on continued rigorous research, including well-designed preclinical studies and clinical trials. These efforts are crucial to refining therapeutic strategies, improving patient outcomes, and expanding the applicability of this innovative approach across diverse cancer types.

## Data Availability

Not applicable.

## Abbreviations

LDRT: Low-Dose Radiation Therapy
HDRT: High-Dose Radiation Therapy
ICIs: Immune Checkpoint Inhibitors
CAR-T: Chimeric Antigen Receptor T cells
TME: Tumor Microenvironment
TIL: Tumor-Infiltrating Lymphocytes
ISV: In-Situ Vaccination
WBRT: Whole-Brain Radiation Therapy
TTField: Tumor Treating Fields
TRT: Targeted Radionuclide Therapy
PD-L1: Programmed Death-Ligand 1
NK: Natural Killer (cells)
CD8+: Cluster of Differentiation 8 Positive (T-cells)
CTLA-4: Cytotoxic T-Lymphocyte Antigen 4
mUM: Metastatic Uveal Melanoma
HCC: Hepatocellular Carcinoma
VEGFA: Vascular Endothelial Growth Factor A
CT: Chemotherapy
CRT: Chemo-Radiation Therapy
PD: Progressive Disease
DFS: Disease-Free Survival
CXCL10/CXCR3: Chemokine Ligand 10 / Chemokine Receptor 3
GM-CSF: Granulocyte-Macrophage Colony-Stimulating Factor
OV: Oncolytic Virus
TCR: T Cell Receptor
CI: Confidence Interval
PD-1: Programmed Cell Death Protein 1
VEGFR: Vascular Endothelial Growth Factor Receptor
IL-12: Interleukin 12
ICAM-1: Intercellular Adhesion Molecule 1
VCAM-1: Vascular Cell Adhesion Molecule 1
IFN-γ: Interferon Gamma
ECM: Extracellular Matrix
MDSC: Myeloid-Derived Suppressor Cells
CAF: Cancer-Associated Fibroblasts
PD1/PD-L1: Programmed Death 1 / Programmed Death Ligand 1
MHC: Major Histocompatibility Complex
